# Suitability of Different Mapping Algorithms for Genome-Wide Polymorphism Scans with Pool-Seq Data

**DOI:** 10.1534/g3.116.034488

**Published:** 2016-09-09

**Authors:** Robert Kofler, Anna Maria Langmüller, Pierre Nouhaud, Kathrin Anna Otte, Christian Schlötterer

**Affiliations:** * Institut für Populationsgenetik, Vetmeduni Vienna, Veterinärplatz 1, 1210 Wien 1210, Austria; † Vienna Graduate School of Population Genetics, 1210, Austria

**Keywords:** Pool-Seq, bioinformatics, Next Generation Sequencing, mapping algorithm, Drosophila

## Abstract

The cost-effectiveness of sequencing pools of individuals (Pool-Seq) provides the basis for the popularity and widespread use of this method for many research questions, ranging from unraveling the genetic basis of complex traits, to the clonal evolution of cancer cells. Because the accuracy of Pool-Seq could be affected by many potential sources of error, several studies have determined, for example, the influence of sequencing technology, the library preparation protocol, and mapping parameters. Nevertheless, the impact of the mapping tools has not yet been evaluated. Using simulated and real Pool-Seq data, we demonstrate a substantial impact of the mapping tools, leading to characteristic false positives in genome-wide scans. The problem of false positives was particularly pronounced when data with different read lengths and insert sizes were compared. Out of 14 evaluated algorithms novoalign, bwa mem and clc4 are most suitable for mapping Pool-Seq data. Nevertheless, no single algorithm is sufficient for avoiding all false positives. We show that the intersection of the results of two mapping algorithms provides a simple, yet effective, strategy to eliminate false positives. We propose that the implementation of a consistent Pool-Seq bioinformatics pipeline, building on the recommendations of this study, can substantially increase the reliability of Pool-Seq results, in particular when libraries generated with different protocols are being compared.

Sequencing pools of individuals (Pool-Seq) is a cost efficient approach to generating genome-wide polymorphism data, which is enjoying increasing popularity (reviewed in Schlötterer *et al.* 2014). For example, Pool-Seq was used to unravel the genetic basis of complex traits (Bastide *et al.* 2013; Cheeseman *et al.* 2015), identify loci contributing to local adaptation (Lamichhaney *et al.* 2012; Turner *et al.* 2010), trace beneficial loci during experimental evolution (Lang *et al.* 2013; Orozco-terWengel *et al.* 2012; Tobler *et al.* 2014), identify positively selected loci in populations (Bergland *et al.* 2014; Kofler *et al.* 2012; Nolte *et al.* 2012), find genes selected during domestication (Axelsson *et al.* 2013; Rubin *et al.* 2010), study the invasion of transposable elements (Kofler *et al.* 2015a), investigate clonal evolution in cancer (Ding *et al.* 2012), and to identify causative mutations in forward genetic screens (Schneeberger *et al.* 2009). With this rapid gain in popularity, it is important to ensure the reliable analysis of Pool-Seq data. Several studies have investigated various aspects that potentially affect the accuracy of Pool-Seq, including the sequencing platform (Rellstab *et al.* 2013), reference genome (Nevado *et al.* 2014), parameters used for aligning reads (Kofler *et al.* 2011a), sequencing depth (Ferretti *et al.* 2013; Kofler and Schlötterer 2014), pool size (Futschik and Schlötterer 2010; Gautier *et al.* 2013), and library preparation protocol (Kofler *et al.* 2015b) used.

However, until now, the impact of the mapping algorithm used for aligning Pool-Seq data has not been studied in sufficient detail. Here, we show that the mapping algorithm can have a profound effect, leading to erroneous signals of allele frequency differences between libraries. We compared systematically the performance of 14 different alignment algorithms using both simulated and real Pool-Seq data. Of the algorithms tested, clc4, novoalign, and bwa mem consistently produced the most reliable results with Pool-Seq data. Nevertheless, no single alignment algorithm avoids all artifacts, but, by intersecting the results of two alignment tools, the vast majority of artifactual outliers can be avoided.

## Material and Methods

### Alignment algorithms

We tested seven semiglobal alignment algorithms, where the entire read is required to match, and seven local alignment algorithms, where only a part of the read needs to match (Table 1). For tools that support semiglobal, as well as local, alignments, we evaluated the suitability of both algorithms (Table 1). We also included gsnap (Wu and Nacu 2010) into our study, despite the fact that this tool was designed for aligning RNA-Seq data (*i.e.*, alignments with large gaps to allow for spliced introns). We also aimed to include gem (Marco-Sola *et al.* 2012), batalign (Lim *et al.* 2015), stampy (Lunter and Goodson 2011), and soap2 (Li *et al.* 2009b) into our study but were not able to run these tools on our computational infrastructure (Mac Pro; batalign: did not respond, gem: compilation failed, stampy: compilation failed due to missing files, soap2: segmentation fault while indexing the reference genome). If possible, we used default parameters for all tools, and deviated from these settings only when deemed necessary to ensure an unbiased comparison of the alignment algorithms (Table 1). With Bowtie2, we set the maximum fragment length of paired ends (–X) to 1500. For bwa, we used version 0.7.4 for the mem and bwasw algorithm, and version 0.6.2 for the aln algorithm. This was necessary as bwa aln 0.7.4 reports a segmentation fault when aligning some data sets (*e.g.*, the *Drosophila simulans* libraries), whereas the mem algorithm was not available for bwa version 0.6.2. For clc4, we interleaved the sequences of the two fastq files (-i), activated the paired end mode (-p), set the orientation of the paired ends to forward followed by backward (fb), and measured the distance between paired ends from start-to-start (ss). As the performance of clc4 is highly sensitive to the provided minimum distance (min) and maximum distance (max) between paired ends, we provided the most suitable setting for each alignment (simulated data, read length 50 and inner distance 100: min=160 max=240, read length 100 and inner distance 100: min=260 max=340, read length 100 and inner distance 300: min=380 max=620; *D. simulans* libraries, read length 76: min=176 max=280, read length 120: min=270 max=390). For mrfast, we used paired end mapping (-pe), provided a minimum fragment size of 10 (−min), a maximum fragment size of 400 (−max; for the simulated data with an inner distance of 300, −max 700 was used), a maximum number of mismatches of 6 (-e), and required that only the best position of a read should be reported (–best). We specified bam as output format (-b) for ngm, and performed a sensitive search (–sensitive; the default is unclear). For novoalign, we provided sam as output (-o SAM), set the quality encoding of fastq files to Sanger (-o STDFQ), required that a random position is reported for ambiguously mapped reads (-r Random), and provided suitable estimates for the insert size (mean) and the SD of the insert size (-i mean SD; simulated data: mean=350 SD=50; *D. simulans* libraries, read length 76: mean=228 sd=52, read length 120: mean=396 SD=110). For segemehl, we set the maximum insert size to 1500 (-I). For gsnap, we used sam as output format (-A sam). Only for the *D. simulans* libraries was the maximum number of allowed mismatches set to 1 (-m 1), as gsnap encountered an error using these data and default settings. (Problem sequence; we iteratively removed five problem sequences but still encountered the error). Alignments with bwa aln were done on a Hadoop cluster (Pandey and Schlötterer, 2013) http://journals.plos.org/plosone/article?id=10.1371%2Fjournal.pone.0072614.

**Table 1:** Overview of the mapping algorithms used in this work

	Mapper	Version	Parameter	Reference
Global	bowtie2(g)	2.2.6	**–**end-to-end*^a^* –X 1500	Langmead and Salzberg 2012
	bwa aln	0.6.2		Li and Durbin 2009
	clc4(g)	4.4.2.133896	**-**a globa**l***^a^* -i -p fb ss min*^b^*, max*^b^*	CLC bio 2015
	mrfast	2.6.1.0	–pe –min 10 –max 400*^b^* –best -e 6	Alkan *et al.* 2010
	ngm(g)	0.4.13	**–**end-to-end*^a^* -b –sensitive	Sedlazeck *et al.* 2013
	novoalign(g)	3.03.2	**-**o FullNW*^a^* -i mean*^b^*, SD*^b^* -F STDFQ -o SAM -r Random	Novocraft 2014
	segemehl	0.2.0-418	-I 1500	Hoffmann *et al.* 2009
Local	bowtie2(l)	2.2.6	**–**local*^a^* –X 1500	Langmead and Salzberg 2012
	bwa sw	0.7.4		Li and Durbin 2010
	bwa mem	0.7.4		Li and Durbin 2009
	clc4(l)	4.4.2.133896	**-**a local*^a^* -i -p fb ss min*^b^*, max*^b^*	CLC bio 2015
	gsnap	2015-11-20	-A sam (-m 1)*^b^*	Wu and Nacu 2010
	ngm(l)	0.4.13	**–**local*^a^* -b –sensitive	Sedlazeck *et al.* 2013
	novoalign(l)	3.03.2	-i mean*^b^*, SD*^b^* -F STDFQ -o SAM -r Random	Novocraft 2014

a Parameters used for selecting semiglobal (g) or local (l) alignments.

b See text for more details.

### Data sets

We tested the performance of the different alignment algorithms using both simulated and real data.

Simulated paired end data were generated for populations having SNPs with known positions and allele frequencies. This was accomplished in four steps. We first obtained the *Drosophila melanogaster* reference chromosome 2R (r6.03; http://flybase.org/), removed all characters other than A, T, C, or G, and extracted the first 2 Mbp. This small subsequence (the chassis) acted as a basis for introducing variants. Second, we generated two modified versions of the chassis: (i) we introduced a SNP with a random, not-reference, allele every 100 bp into the chassis (⇒chassis with SNPs), and (ii) we introduced an indel at a random position with a random Poisson distributed length (λ=1; zero length indels were discarded and Poisson sampling was repeated; insertions had a random sequence), between all pairs of adjacent SNPs into the chassis with SNPs (⇒chassis with SNPs and indels). Third, we generated two sequences serving as templates for simulating paired ends: one consisting of the chassis and the chassis with SNPs (Figure 2A), and another consisting of the chassis and the chassis with SNPs and indels (Figure 2B). Finally, uniformly distributed paired end reads (equal 5′ distance between consecutive paired ends; uniform base quality of 40) were simulated from these template sequences (Figure 2C). Note that SNPs identified from these data have known positions (each 100 bp) and known allele frequencies (f=0.5). Paired end reads were simulated with SimulaTE (https://sourceforge.net/projects/simulates/; R. V. Pandey *et al.* personal communications), and the number of reads was selected such that a genomic coverage of 200 resulted (*generate-reads_paired-end-uniformdistribution.py*; ∼2 million paired ends for a read length of 100, and 4 million for a read length of 50).

We tested the performance of the different alignment algorithms for real data using paired end reads from a *D. simulans* population that was collected in 2008 in Northern Portugal (Póvoa de Varzim; provided by P. Orozco-terWengel). We established 250 isofemale lines from the population, used one female from each isofemale line, and extracted genomic DNA from the pooled flies as described (Orozco-terWengel *et al.* 2012). From this DNA, we generated two Illumina sequencing libraries. The first was prepared using the Paired-End DNA Sample Preparation Kit (Illumina, San Diego, CA) following fragmentation of the DNA using a nebulizer, and size selection using an agarose gel. The library was sequenced on two lanes of an Illumina GAIIx, resulting in 14.3 and 24.7 million 2 × 76 bp paired end reads after trimming [median insert size 232 bp; SD of the insert size 25 bp; estimated with Picard v1.128 (http://picard.sourceforge.net) after mapping the reads with bwa aln (0.6.2) (Li and Durbin 2009)].

The second library was prepared with barcoded adapters using a protocol based on the NEBNextDNA Library Prep Master Mix Set reagents (E6040L) following shearing pooled genomic DNA with a Covaris S2 device (Covaris, Woburn, MA), and size selection with AMPureXP beads (Beckman Coulter, Brea, CA). The library was sequenced on one lane of an Illumina HiSeq 2500 using 2 × 120 bp reads (median insert size 396 bp; SD of the insert size 110 bp; 84.5 million paired end reads after trimming).

The quality encoding of all reads was converted to Sanger (offset = 33), and low quality regions of reads were trimmed with ReadTools (https://github.com/magicDGS/ReadTools–disable-zipped-output–minimum-length50–no-5p-trim–quality-threshold18; per default the quality is converted to Sanger encoding). ReadTools provides a fast implementation of the trimming algorithm described in Kofler *et al.* (2011a).

We tested whether intersecting of mappers preserves the targets of selection using the data published by Martins *et al.* (2014). We obtained Illumina paired end data (2 × 100 bp) for four populations infected with C-virus for 20 generations (VirSys; accession numbers ERS409784-ERS409787), and for four control populations (ContSys; accession numbers ERS409780-ERS409783).

### Data analysis

The simulated reads were mapped to the chassis (see above), the *D. simulans* libraries were mapped to the reference genome of strain M252 (Palmieri *et al.* 2015) (v1.1; we included the sequences of *Lactobacillus brevis*, *Acetobacter pasteurianus*, and two Wolbachia strains; GenBank accession numbers CP000416.1, AP011170.1, AE017196.1, and CP001391.1), and the data from Martins *et al.* (2014) were mapped to the reference genome of *D. melanogaster* (v6.03; we again included the sequences of *L. brevis*, *A. pasteurianus*, and two Wolbachia strains). If not mentioned otherwise, mapped reads were filtered for mapping quality (-q 20), and proper pairs (-f 0x002 -F 0x004 -F 0x008; except for the analysis of single end reads), with samtools (v1.2) (Li *et al.* 2009a). Mapped reads were converted to mpileup files with samtools (v1.2), and the parameters -B -Q 0. SNPs were called using a minimum allele count of 2. The number of true SNPs (every 100th position), the number of false SNPs (not at every 100th position), the frequency of the reference allele (only for true positive SNPs), and the number of extreme outlier SNPs (where the estimated allele frequency deviates by > 0.4 from the true frequency 0.5), were computed using custom Python scripts (*snp-caller.py*, *stat-snp.py*). For computing allele frequency differences between samples, mpileup files were created with samtools (v1.2; -B -Q 0), the mpileup files were converted to sync files with PoPoolation2 [revision 196; *mpileup2sync.jar* –fastq-type sanger; the minimum quality (–min-qual) was set to 0 for simulated reads, and to 20 for *D. simulans* libraries; (Kofler *et al.* 2011b)], and $F_{\mathrm{ST}}$ or Fisher exact test *P*-values (−log10 transformed) were computed with PoPoolation2 (revision 196; *fst-sliding.pl* –min-count 2 –min-coverage 10 –max-coverage 500 –window-size 1 –step-size 1 –suppress-noninformative –pool-size 400 –min-covered-fraction 1.0; *fisher-test.pl* –min-count 2 –min-coverage 10 –max-coverage 500 –window-size 1 –step-size 1 –min-covered-fraction 1.0). The outlier quantiles of $F_{\mathrm{ST}}$ and *P*-values (Fisher exact test; −log10(*P*-values)] were calculated with Python scripts (*fst-fractionwise.py*). Results of two mapping algorithm were intersected with a custom script (*merge-teststat.py*).

We evaluated the performance of the following quality filtering methods: (i) a minimum mapping quality of 40 (using samtools v1.2; parameters -B -Q 0 -q 40), (ii) a minimum allele count of 10 (using PoPoolation2; revision 196; *fisher-test.pl* –min-count 10 –min-coverage 10 –max-coverage 500 –window-size 1 –step-size 1 –min-covered-fraction 1.0), (iii) a minimum base quality of 30 (using PoPoolation2; *mpileup2sync.jar* –min-qual 30), (iv) the 10% of the SNPs with the most pronounced strand bias were removed (strand-bias was computed as $\mathrm{SB}=\left|f_{\mathrm{fwd}}-0.5\right|,$ where $f_{\mathrm{fwd}}$ is the frequency of reads mapping to the forward strand at a given site, irrespective of the allele), and (v) sites not called by FreeBayes were removed [FreeBayes v1.0.2-6-g3ce827d (Garrison and Marth 2012); parameters -X -K -C 1 -F 0.01 (input data are pooled, ignore multi-allele SNPs, minimum allele count of 1, minimum allele frequency of 0.01)].

Differentiation between evolved and control populations for the data from Martins *et al.* (2014) was assessed with the Cochran-Mantel-Haenszel test (CMH) implemented in PoPoolation2 (Kofler *et al.* 2011b) (parameters: –min-count 2 –min-coverage 10 –max-coverage 500).

Aligned reads were inspected visually using IGV (Thorvaldsdóttir *et al.* 2013), and statistical analyses was performed using the R programming language (R Core Team 2012).

### Data availability

The short reads have been made available at the European Nucleotide Archive (ENA; http://www.ebi.ac.uk/ena; PRJEB13602), and the scripts used in this work, as well as the simulated reads, are available at Dryad (http://datadryad.org/doi:10.5061/dryad.2g3s4).

## Results

**Figure 1:**
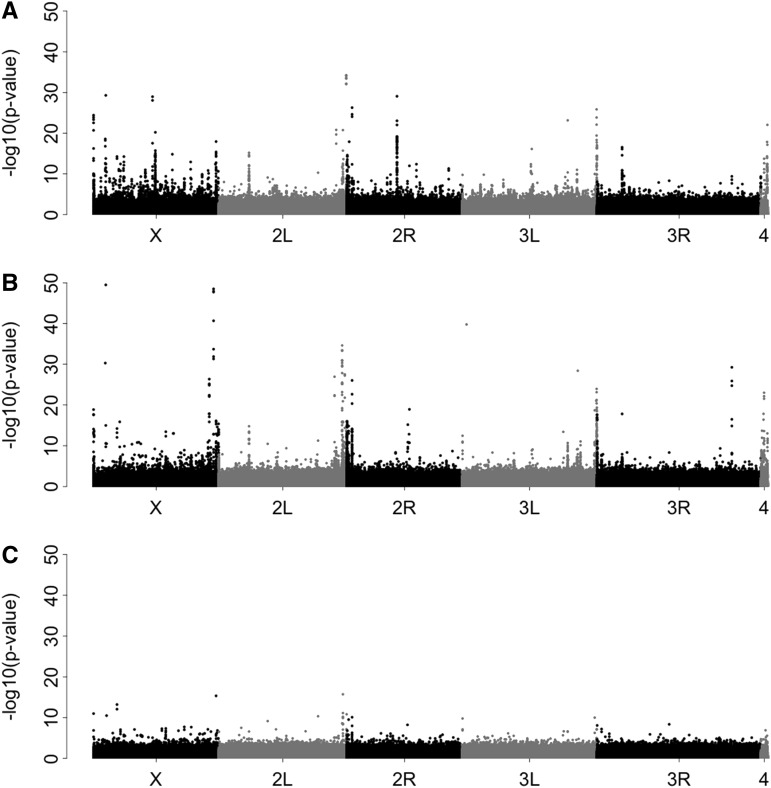
Manhattan plots indicating the significance of allele frequency differences between Pool-Seq libraries when the same genomic DNA is sequenced. Two Illumina paired-end sequencing libraries with different read length and insert sizes were prepared from a pool of 250 *D. simulans* individuals. Reads were mapped to the reference genome, and the significance of differences in allele frequencies between the two libraries were computed (Fisher’s exact test). Although no significant allele frequency differences were expected, we found pronounced outlier peaks using bwa aln (A) or novoalign(g) (B) for mapping the reads. Importantly, outlier peaks found with these two alignment algorithms are at different genomic sites. Hence, intersecting the results of these two algorithms by plotting the lowest *P*-value obtained at each site removes the vast majority of outlier peaks (C).

Genome-wide polymorphism scans with Pool-Seq data are becoming increasingly used in population genomic research. Typically, these studies use genome-wide Pool-Seq data to identify marked outlier loci in pairwise comparisons between population samples. For example, loci contributing to local adaptation are identified by significantly different allele frequencies between populations (Lamichhaney *et al.* 2012; Turner *et al.* 2010). This focus on outlier loci makes genome-wide scans susceptible to technical problems that could generate outlier artifacts. We found that the mapping algorithms for aligning Pool-Seq data may be an important source of outlier artifacts (Figure 1). Comparing allele frequencies between two Pool-Seq libraries prepared from identical genomic DNA, but with different insert size and read length, we found a substantial number of outlier loci, despite the fact that no differences between the libraries were expected (Figure 1, A and B).

To overcome this problem, we set out to identify alignment algorithms that are most suitable for genome-wide outlier scans using Pool-Seq data. We tested seven semiglobal alignment algorithms, where the entire read is required to match [bowtie2(g), bwa aln, clc4(g), mrfast, ngm(g), novoalign(g), and segemehl], and seven local alignment algorithms, where only a part of the read needs to match [bwa sw, bwa mem, clc4(l), gsnap, ngm(l), and novoalign(l); for an overview see Table 1] (Alkan *et al.* 2010; CLC bio 2015; Hoffmann *et al.* 2009; Langmead and Salzberg 2012; Li and Durbin, 2009, 2010; Novocraft 2014; Sedlazeck *et al.* 2013; Wu and Nacu 2010). With several tools, like ngm or bowtie2, supporting both semiglobal and local alignments, we indicate the pertinent algorithm in brackets [*e.g.*: ngm(g): semiglobal alignment, ngm(l): local alignment].

**Figure 2:**
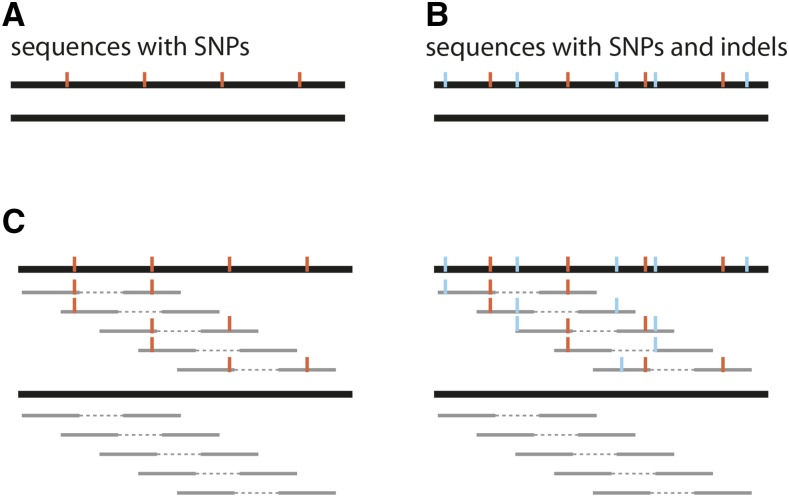
Overview of simulated Pool-Seq data sets. Based on a 2 Mbp region of *D. melanogaster* chromosome 2R, we simulated a pair of sequences, with one sequence having a SNP (red) every 100 bp (A), and a pair of sequences with one sequence having, in addition to the SNPs, an indel (blue) with random position and length between adjacent SNPs (B). Using these sequences as templates, we simulated uniformly distributed paired ends (gray; C) resulting in SNPs with known positions and frequency (f = 0.5).

We first tested the overall performance of the alignment algorithm using simulated data sets. We generated template sequences with SNPs and indels (random position and length) at known positions, and then simulated uniformly distributed paired ends from these templates such that true SNPs are spaced exactly 100 bp apart, and have a population frequency of 0.5 (Figure 2). Note that indels are in linkage disequilibrium with SNPs to identify biased allele frequency estimates resulting from mapping of reads with indels.

We evaluated the mapping algorithms with three different paired end data sets: (i) a data set representing optimal conditions (2 × 100 bp paired ends; insert size 100±0 bp; error rate of 0%; no indels; Figure 2A), (ii) a data set with indels and variation of the distance between paired ends (2 × 100 bp paired ends; insert size 100±40 bp; error rate of 0%; indels; Figure 2B), and a dataset with indels and a high error rate (polymorphism) of 5% (2 × 100 bp paired ends with an insert size of 100±0 bp; error rate of 5%; indels; Figure 2B). For all data sets, a coverage of 200 per site was targeted (∼2 million paired ends per data set). We evaluated the performance of the mapping algorithms based on two criteria: the number of true positive SNPs, and the number of extreme outlier loci with highly inaccurate allele frequency estimates (f≥0.9 or f≤0.1; frequency should be f=0.5).

We compared the performance of the mapping algorithms with and without filtering for quality criteria, such as paired end reads and mapping quality [≥20; a low mapping quality suggest that the read is ambiguously mapped (Li *et al.* 2008)], and found that filtering consistently leads to reduced numbers of false positive SNPs and more accurate allele frequency estimates (Supplemental Material, Table S1; note that, in the absence of sequencing errors, false positive SNPs are an artifact of the alignment). This observation is in agreement with previous work showing that quality filtering can reduce the number of false positive SNPs (Li *et al.* 2008). We note, however, that quality filtering also leads to fewer true positive SNPs (Table S1).

**Figure 3:**
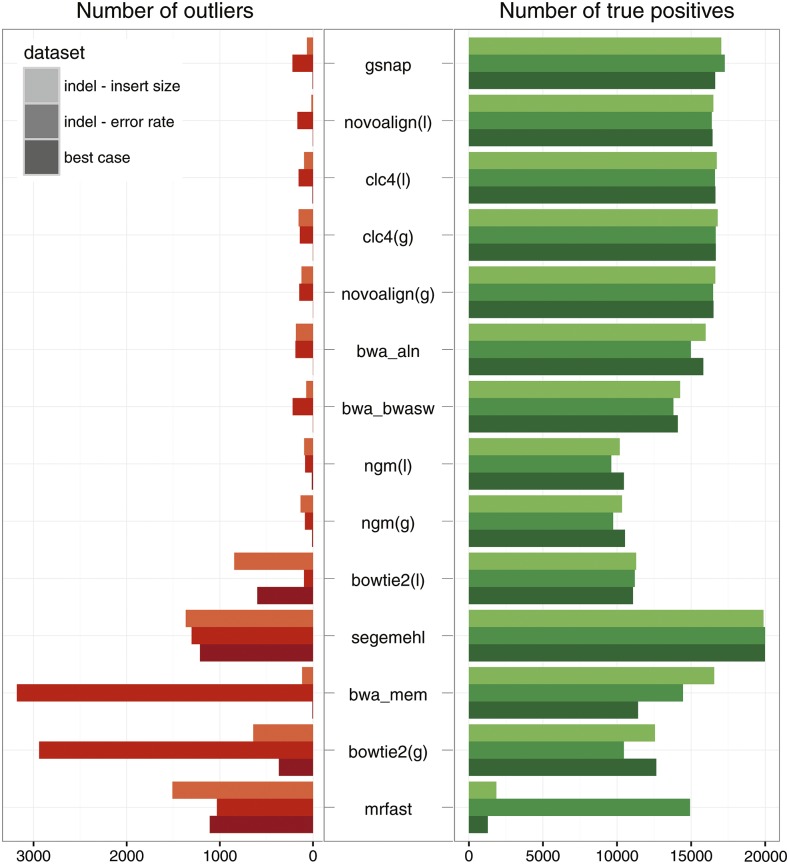
Suitability of mapping algorithms for performing genome wide polymorphism scans with Pool-Seq data. Ideally, a mapping algorithm should enable the identification of all truly positive SNPs (green; 19.999 were simulated), while avoiding the identification of extreme outlier SNPs, with highly inaccurate allele frequency estimates (red; f>0.9 or f<0.1). Algorithms are sorted according to performance, with the best performing algorithm shown at the top (maximizing the true positives, and minimizing the number of outliers). We tested the algorithm with three different data sets. Best case: 2 × 100 bp paired ends with an insert size of 100±0 bp, indel - insert size: 2 × 100 bp paired ends with an insert size of 100±40 bp, and indels between the SNPs, indel - error rate: 2 × 100 bp paired ends with an insert size of 100±0 bp, indels between the SNPs and an error rate of 5%.

Quality filtering also affected the coverage distribution. Fewer sites had a higher coverage than simulated in filtered data (Figure S1), which is likely due to smaller numbers of ambiguously mapped reads that stochastically accumulate in some genomic regions. For mrfast, quality filtering resulted in a severe shift of the coverage distribution, halving the average coverage (Figure S1). The distribution of mapping qualities differed between mapping algorithms (Figure S2), which is likely due to distinct algorithms for computing mapping qualities. Since the accuracy of allele frequency estimates was substantially better for filtered data sets, we rely on quality filtered reads for the remaining manuscript. Summarizing the results for all three simulated data sets, we found that gsnap, novoalign(l), clc4(l), and clc4(g) showed the best performance, while mrfast, bowtie2(g), and bwa mem showed the worst (Figure 3 and Table S2; for results with unfiltered data, see Table S3). The average reference allele frequency of most alignment algorithms was above 0.5 indicating a bias toward the reference allele [Table S2; see also Degner *et al.* 2009; Kofler *et al.* 2011a]. After quality filtering, mrfast had a substantial bias against the reference allele (Table S3).

**Figure 4:**
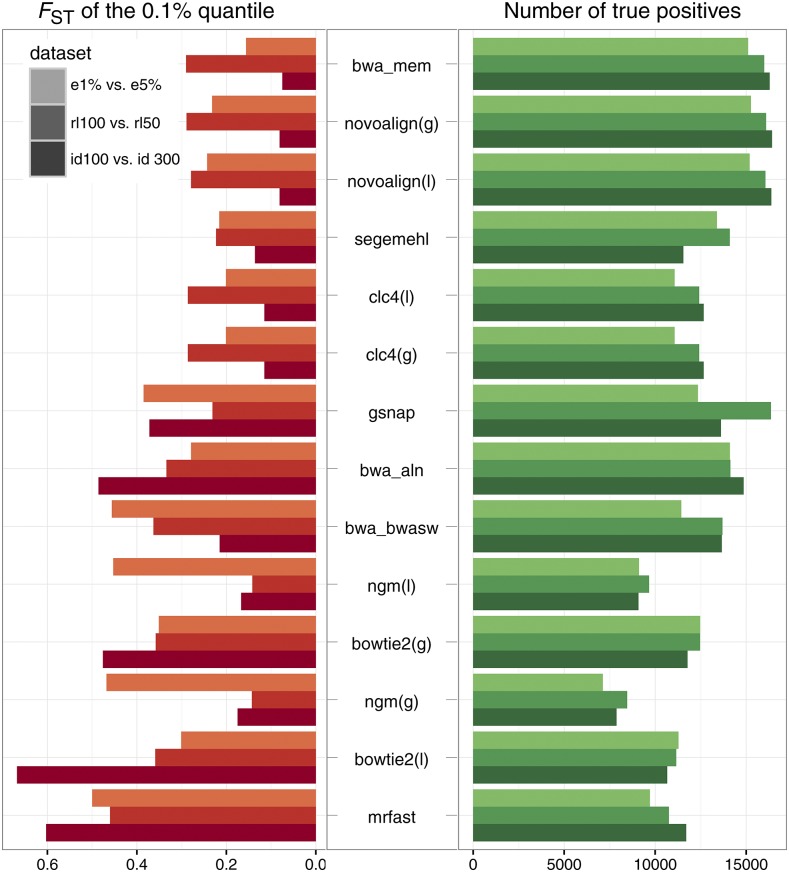
Comparison of allele frequency differences between simulated Pool-Seq data sets with different mapping algorithms. We simulated different paired end Pool-Seq libraries, mapped the reads, and compared the allele frequencies between the libraries using $F_{\mathrm{ST}}.$ With this procedure, we evaluated the sensitivity of the alignment algorithm to differences in the distance between paired ends (id), differences in the read length (rl), and differences in the error rates (e). As all libraries were derived from identical template sequences (templates with SNPs and indels), no significant allele frequency differences were expected ($F_{\mathrm{ST}}=0$). We estimated the number of truly positive SNPs for which allele frequencies could be compared (TP), and the lowest $F_{\mathrm{ST}}$-values in the 0.1% quantile with the most differentiated SNPs. Algorithms are sorted according to performance, with the best performing algorithm shown at the top (maximizing the true positives, and minimizing the $F_{\mathrm{ST}}$ in the outlier quantile). id100, rl100, e1%: 2 × 100 bp paired ends, insert size 100±20 bp, error rate 1%; id300: 2 × 100 bp paired ends, insert size 300±60 bp, error rate 1%; rl50: 2 × 50 bp paired ends, insert size 100±20 bp, error rate 1%; e5%: 2 × 100 bp paired ends, insert size 100±20 bp, error rate 5%.

Next, we compared allele frequency estimates between samples—an approach that is typically used to identify loci responsible for local adaption. We investigated the sensitivity of the alignment algorithm to (i) differences of the inner distance between paired ends (inner distances 100±20 bp *vs.* 300±60 bp), (ii) differences in read length (read length 100 *vs.* 50 bp) and (iii) differences in the error rates (error rates 1 *vs.* 5%). Uniformly distributed paired ends were simulated from the template sequences having SNPs and indels (Figure 2B). Allele frequency differences between samples were measured using $F_{\mathrm{ST}}.$ Values of $F_{\mathrm{ST}}$ range from 0 to 1, where 0 indicates no differentiation between samples (populations), and 1 indicates complete differentiation (fixation for alternative alleles) (Hartl and Clark 1997). As all paired ends have a uniform genomic distribution, and were derived from the same template sequences, only small allele frequency differences are expected between samples. A perfect alignment algorithm would detect all positive SNPs (TP=19.999), and yield a low $F_{\mathrm{ST}}$ for all SNPs ($F_{\mathrm{ST}}=0$). Based on the simulated data bwa sw mem, novoalign(g), and novoalign(l) showed the best performance, whereas mrfast, bowtie2(l), and ngm(g) performed worst (Figure 4 and Table S4; for allele frequency differences with false positive SNPs, see Table S5). We noted substantial allele frequency differences when the same data were mapped as paired end and as single end reads, and then compared against each other (Table S7). ngm(g) and ngm(l) were most suitable for such comparisons between paired and single end reads.

**Figure 5:**
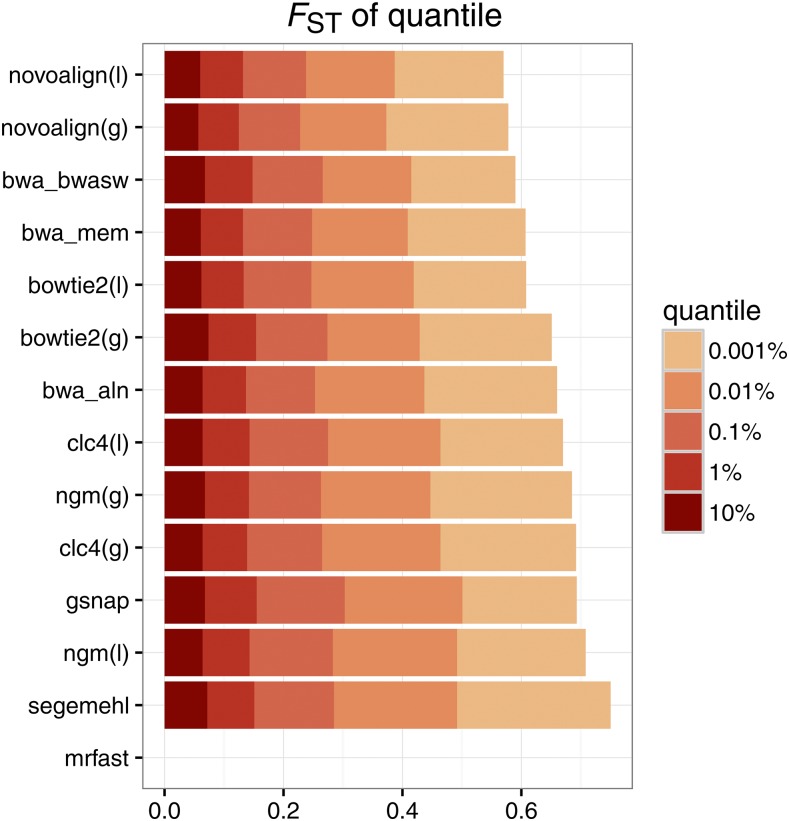
Comparison of allele frequency differences between real Pool-Seq data sets with different mapping algorithms. We compared allele frequencies between two paired end libraries with different read length and insert size that were prepared from the same genomic DNA (pooled *D. simulans* flies). We determined the lowest $F_{\mathrm{ST}}$-values in different quantiles with the most differentiated SNPs. Algorithms are sorted according to performance, with best the performing algorithm shown at the top (minimizing the $F_{\mathrm{ST}}$ in the 0.001% outlier quantile). mrfast generated an invalid output file with these data (an uniform read length was reported despite these reads having varying read lengths).

Simulated data may not capture all the properties of real data, such as reads having different lengths (after trimming), variable base qualities along reads, and biases in sequencing errors. Therefore, we also evaluated the performance of different alignment algorithms based on $F_{\mathrm{ST}}$ between samples using real data. We used two libraries with different read length and insert size prepared from the same genomic DNA (library 1: 2 × 76 bp paired ends, median insert size = 232 bp; library 2: 2 × 120 bp paired ends, median insert size = 396; both prepared from pooled *D. simulans* flies; see *Materials and Methods*), trimmed low quality regions from the 3′-ends of reads, and compared allele frequency differences between the samples using $F_{\mathrm{ST}}.$ As both libraries were prepared from the same genomic DNA, only small allele frequency differences were expected between the samples ($F_{\mathrm{ST}}=0$). Novoalign(l), novoalign(g), and bwa sw showed the best performance ,while mrfast, segemehl, and ngm(l) performed worst (Figure 5).

In summary, when comparing the results of the previous evaluations, we conclude that novoalign(l), novoalign(g), clc4(l), and bwa mem are the most suitable alignment algorithm for Pool-Seq data, whereas mrfast, ngm(l), ngm(g), and bowtie2(g) did not perform as well (Table 2).

**Table 2:** Comparison of alignment algorithms for Pool-Seq data: summary across data sets

Algorithm	Poly.	$F_{\mathrm{ST}}$-sim.	$F_{\mathrm{ST}}$-real	Rank-sum
novoalign(l)	2	3	1	6
novoalign(g)	5	2	2	9
clc4(l)	3	5	8	16
bwa mem*^a^*	12	1	4	17
bwa bwasw*^a^*	7	9	3	19
gsnap*^a^*	1	7	11	19
clc4(g)	4	6	10	20
bwa aln*^a^*	6	8	7	21
segemehl*^a^*	11	4	13	28
bowtie2(l)*^a^*	10	13	5	28
bowtie2(g)*^a^*	13	11	6	30
ngm(g)*^a^*	9	12	9	30
ngm(l)*^a^*	8	10	12	30
mrfast*^a^*	14	14	14	42

Ranks of the algorithm in the previous evaluations are shown: overall suitability (poly: Figure 2), allele frequency differences using simulated data ($F_{\mathrm{ST}}$-sim.: Figure 2), and allele frequency differences using real data ($F_{\mathrm{ST}}$-real: Figure 2). Algorithms are sorted according to performance with best the performing algorithm shown at the top (minimizing the rank-sum).

a Freely available algorithm.

Despite novoalign(g) being one of the most suitable algorithms for Pool-Seq data, a substantial number of artifactual outlier peaks can still be found when comparing the allele frequency between the *D. simulans* libraries (Figure 1). The comparison of different mappers indicated that outlier artifacts are frequently specific to the alignment algorithm (Figure 1, Figure S3, and Figure S4). We reasoned therefore that an intersection of two mappers, recording for every SNP only the least significant result found by any mapper, could overcome this problem. Intersecting the results of bwa and novoalign (Figure 1, A and B), the number of outlier peaks could be substantially reduced (Figure 1C). We tested whether intersecting the results of two mappers results in a more efficient removal of outlier peaks than quality filtering approaches. We evaluated the impact of filtering for (i) mapping quality, (ii) minor allele count, (iii) base quality, (iv) strand-bias, and (v) SNPs called by FreeBayes, a SNP caller well-suited for Pool-Seq data (Garrison and Marth 2012). We found that, of the approaches tested, intersecting the results of two mappers led to the most pronounced reduction of outlier peaks, while the vast majority of the SNPs were retained (Figure S10).

We also tested whether intersecting the results of different mappers preserves the targets of selection using data from an experimental evolution study for C-virus resistance in *D. melanogaster* (Martins *et al.* 2014), and found that the most differentiated loci identified by Martins *et al.* (2014) were retained (Figure S5). Hence, intersecting the results of different mappers is an efficient strategy for minimizing the number of artifacts, while preserving the targets of selection.

To identify the most suitable combination of mapping algorithms, we used the data from the pooled *D. simulans* flies, computed all pairwise intersections of the algorithms, and benchmarked them using the number of SNPs and the 0.001% quantile of most differentiated SNPs (Table S8). ngm(l) combined with bowtie2(g) yielded the least pronounced outlier peaks, with ∼4.15 million shared SNPs [Table S8; for Manhattan plots see Figure S6]. We note, however, that the best combination of alignment algorithms depends on the threshold—with the 0.01% quantile, novoalign(l) and bowtie2(g) are the best combination (Table S9 and Figure S7).

### Conclusions

Here, we performed a comprehensive analysis of different alignment algorithms for Pool-Seq data. The evaluation of alignment algorithms is complicated by several issues. First, mapping quality is computed differently between algorithms (Figure S2). Thus, the fraction of reads filtered by requiring a certain minimum quality (we used 20) varies among the alignment tools. The fraction of filtered reads will affect both the number of identified true positive SNPs, and the accuracy of the allele frequency estimates; more mapped reads result in a higher number of true SNPs, but the number of ambiguously mapped reads is also increased, which distorts allele frequency estimates. The tradeoff between optimizing the recovery of true SNPs and accuracy of the allele frequency estimates is particularly pronounced for segemehl: no reads could be quality filtered since all reads have a mapping quality of 255, resulting in the highest number of true positive SNPs but poor allele frequency estimates (Table S2). Despite this complication, we considered quality filtering of reads essential, as this substantially improves allele frequency estimates from Pool-Seq data (for unfiltered results, see Table S3). Interestingly, the best performing algorithms (*e.g.*, novoalign and clc) identified the highest number of true positive SNPs, and yielded the most accurate allele frequency estimates (Table S2), which suggests that the superior performance of these tools is robust with respect to the tradeoff introduced by quality filtering. Furthermore, we found that the top performing algorithm consistently had the highest ratio of true positive SNPs to false positive SNPs, irrespective of the mapping quality threshold used (Figure S11).

The choice of alignment parameters is a challenge for the comparison of different mapping algorithms. Whenever feasible, we used default parameters, and modified them only when we considered it necessary to ensure an unbiased comparison (*e.g.*, when the error rate exceeded the number of allowed mismatches, or when the insert size was larger than the maximum insert size; see *Materials and Methods*). We note, however, that the performance of each of these algorithms may be improved by fine-tuning the parameters. For example, the performance of bwa aln was substantially improved by using parameters optimized for Pool-Seq data (Kofler *et al.* 2011a) (Table S10). While the optimization of mapping parameters for all 14 algorithms is clearly beyond the scope of this manuscript, we made all data, including the simulated ones, publicly available to allow testing of the performance of different mappers and parameters with these data sets.

We did not consider low frequency alleles for the simulated data for three reasons: (1) mapping errors will have the strongest effect with balanced allele frequencies; (2) the identification of low frequency variants in real Pool-seq data are challenging, as sequencing errors can not be reliably distinguished from base substitutions (Schlötterer *et al.*, 2014); and (3) low frequency variants will not significantly contribute to any measure of sample differentiation.

Out of the 14 algorithms tested, clc4(g), novoalign(g), bwa mem, clc4(l), and novoalign(l) are the most suitable for Pool-Seq data. The superior performance of novoalign is in agreement with previous work, which found that novoalign yields highly accurate alignments and SNP calls (Bao *et al.* 2014; Li and Homer 2010; Nielsen *et al.* 2011).

The most striking influence of different alignment algorithms was noted for experimental data differing in insert size and read length. Comparing different libraries from the same genomic DNA, we identified substantial outliers, some of them clustering in peaks, which indicate allele frequency differences at multiple neighboring sites. Since such peaks are a typical signal in genome-wide outlier scans, such as Pool-genome-wide association (GWAS) or evolve and resequence (E&R) studies, these artifacts may lead to false conclusions. Similar artifacts were also seen when the data were mapped as single reads (Figure S8), suggesting that this is not an artifact of paired end mapping. Assuming that true allele frequency differences between samples should be identified with most alignment tools, whereas artifacts should be found with only a few algorithms, we propose intersecting multiple alignment algorithms. We noticed a clear improvement when intersecting two alignment algorithms, but, depending on the evaluation criteria, different pairs of algorithms perform best. These results are consistent with other studies, which also found that the combination of mapping algorithms and/or variant calling pipelines may yield superior results (Bao *et al.* 2014; Field *et al.* 2015; O’Rawe *et al.* 2013).

Our approach to intersect algorithms is based on the least significant allele frequency differences between two samples. It is straightforward to extend this approach to studies that rely on multiple samples, such as replicated Pool-GWAS experiments or E&R studies (for example, see Figure S5), provided that it is feasible to collapse allele frequency differences between multiple samples into a single representative measure [*e.g.* *P*-value from a cmh-test (Orozco-terWengel *et al.* 2012)]. In this case, again the least significant value found by any mapper may be used. However, this strategy cannot be applied to Pool-Seq data from single populations (*e.g.*, Asgharian *et al.* 2015; Boitard *et al.* 2013; Nolte *et al.* 2012). One possibility to avoid mapping artifacts for single population Pool-Seq data may be to filter SNPs with incongruent allele frequency estimates among multiple mappers. Given that most artifacts were observed when libraries with different insert sizes and read lengths were compared (cf. Figure 1 and Figure S9), we recommend using a single consistent sequencing strategy for all Pool-Seq libraries, whenever possible. We additionally propose to use a single consistent mapping pipeline for all Pool-Seq data, as mixing samples aligned with different tools, algorithms, parameters, or even versions, of the same tool, leads to elevated levels of outlier peaks (Table S10).

## Supplementary Material

Supplemental Material
