# Supplementary material for: Suitability of Different Mapping Algorithms for Genome-Wide Polymorphism Scans with Pool-Seq Data
Source: G3 (Bethesda). 2016 Sep 9;6(11):3507–15. doi: 10.1534/g3.116.034488 (PMC5100849; doi:10.1534/g3.116.034488)
Supplement: Supplemental Material [file supp_6_11_3507__index.html]

Suitability of Different Mapping Algorithms for Genome-Wide Polymorphism Scans with Pool-Seq Data — Supplemental Material 

# Suitability of Different Mapping Algorithms for Genome-Wide Polymorphism Scans with Pool-Seq Data

## Supplemental Material for Kohler *et al.*, 2016

**Files in this Data Supplement:**

- Figure S1 - Coverage distribution for different mappers, before (top) and after (bottom) quality filtering (proper pairs and a mapping quality ≥ 20). (.pdf, 47 KB)
- Figure S10 - Performance of different quality filtering approaches. (.pdf, 25 KB)
- Figure S11 - True positives (TP) compared to false positive (FP) SNPs dependent on the mapping quality threshold for the different alignment algorithms. (.pdf, 150 KB)
- Table S1 - Effect of quality filtering. (.pdf, 22 KB)
- Table S2 - Suitability of mapping algorithms for performing genome wide polymorphism scans with Pool-Seq data. (.pdf, 26 KB)
- Table S3 - Suitability of different mapping algorithms for performing genome wide polymorphism scans with unfiltered Pool-Seq data. (.pdf, 26 KB)
- Table S4 - Comparison of allele frequency differences between simulated Pool-Seq data sets with different mapping algorithms. (.pdf, 25 KB)
- Table S5 - Comparison of allele frequency differences for false positive SNPs (FP) between simulated Pool-Seq data sets with different mapping algorithms. (.pdf, 20 KB)
- Table S6 - Comparison of allele frequency differences between real Pool-Seq data sets with different mapping algorithms. (.pdf, 19 KB)
- Table S7 - Comparison of allele frequency differences using the same sequencing reads, but mapped as paired ends and single ends. (.pdf, 17 KB)
- Table S8 - Reduction of mapping artifacts by the intersection of mapping algorithms using the 0.001% quantile. (.pdf, 26 KB)
- Figure S2 - Mapping quality distribution for different mappers. (.pdf, 20 KB)
- Table S9 - Reduction of mapping artifacts by the intersection of mapping algorithms using the 0.01% quantile. (.pdf, 25 KB)
- Figure S5 - Manhattan plots showing the dierentiation between a *D. melanogaster* population inoculated with C-virus for 20 generations and a control population (data from Martins et al., 2014). (.pdf, 144 KB)
- Table S10 - Comparing allele frequency estimates between samples mapped with dierent alignment algorithms. (.pdf, 27 KB)
- Figure S3 - Example of an outlier peak identified with bwa but not with novoalign. (.pdf, 31 KB)
- Figure S4 - Example of an outlier peak identified with novoalign but not with bwa. (.pdf, 34 KB)
- Figure S6 - Manhattan plots for reads aligned with bowtie2(g) (top panel), ngm(l) (middle panel) and for the intersection of the mapping results (bottom panel). (.pdf, 100 KB)
- Figure S7 - Manhattan plots for reads aligned with bowtie2(g) (top panel), novoalign(l) (middle panel) and for the intersection of the mapping results (bottom panel). (.pdf, 100 KB)
- Figure S8 - Manhattan plots for reads aligned as single ends with bwa aln (top panel), novoalign(g) (middle panel) and for the intersection of the mapping results (bottom panel). (.pdf, 127 KB)
- Figure S9 - Manhattan plots for a comparison of two of paired end data sets having similar read length and insert size. (.pdf, 75 KB)
