# Supplementary material for: Suitability of Different Mapping Algorithms for Genome-Wide Polymorphism Scans with Pool-Seq Data
Source: G3 (Bethesda). 2016 Sep 9;6(11):3507–15. doi: 10.1534/g3.116.034488 (PMC5100849; doi:10.1534/g3.116.034488)
Supplement: Supplemental Material [file supp_g3.116.034488_FigureS10.pdf]

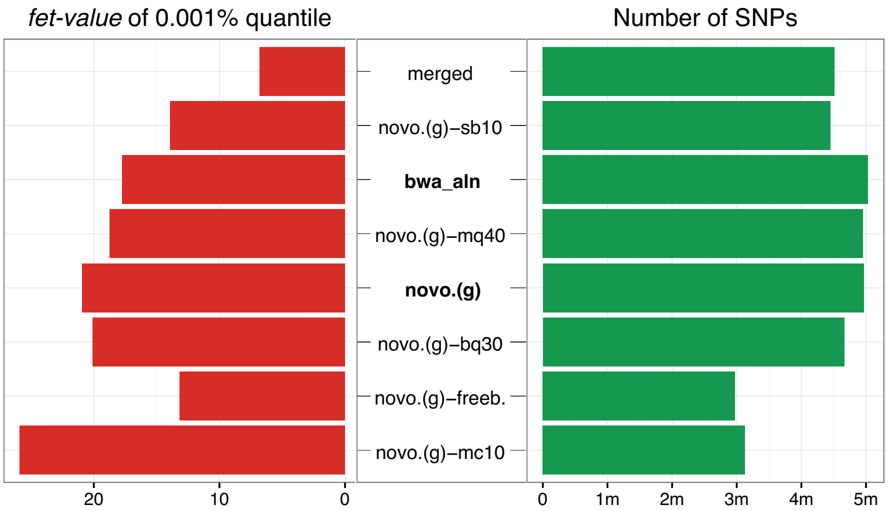

Figure 10: Performance of different quality filtering approaches. Two Illumina paired end data sets with different insert sizes and read length were derived from pooled genomic DNA (natural *D. simulans* population) and mapped to the reference genome using bwa aln and novoalign(g) [novo.(g)]. Allele frequency differences between the libraries were computed using Fisher's exact test [-log(p-value) = *fet-value*]. Ideally, quality filtering should reduce the size of outlier peaks (measured as lowest *fet-value* among the 0.001% most differentiated SNPs; red), but retain most SNPs (green). In addition to intersecting the results of novoalign(g) and bwa aln (merged), we evaluated the following quality filtering approaches: a minimum mapping quality of 40 (mq40), a minimum allele count of 10 (mc10), a minimum base quality of 30 (bc30), removing the 10% of SNPs with the most pronounced strand-bias (sb10) and removing SNPs not identified by FreeBayes (freeb.). Unfiltered data are marked bold. Approaches are sorted according to performance, with the best performing approach shown at the top (minimizing outlier peaks and maximizing retained SNPs).
