# Supplementary material for: Suitability of Different Mapping Algorithms for Genome-Wide Polymorphism Scans with Pool-Seq Data
Source: G3 (Bethesda). 2016 Sep 9;6(11):3507–15. doi: 10.1534/g3.116.034488 (PMC5100849; doi:10.1534/g3.116.034488)

Figure 11: True positives (TP) compared to false positive (FP) SNPs dependent on the mapping quality threshold for the different alignment algorithms. Uniformly distributed paired end reads (2x100bp) with an insert size of 100±40bp and an error rate of 0% were simulated from sequences with SNPs every 100bp and indels between the SNPs, the reads were aligned and SNPs were identified. Note since we simulated an error rate of 0% all false positive SNPs are an artifact of the alignments.

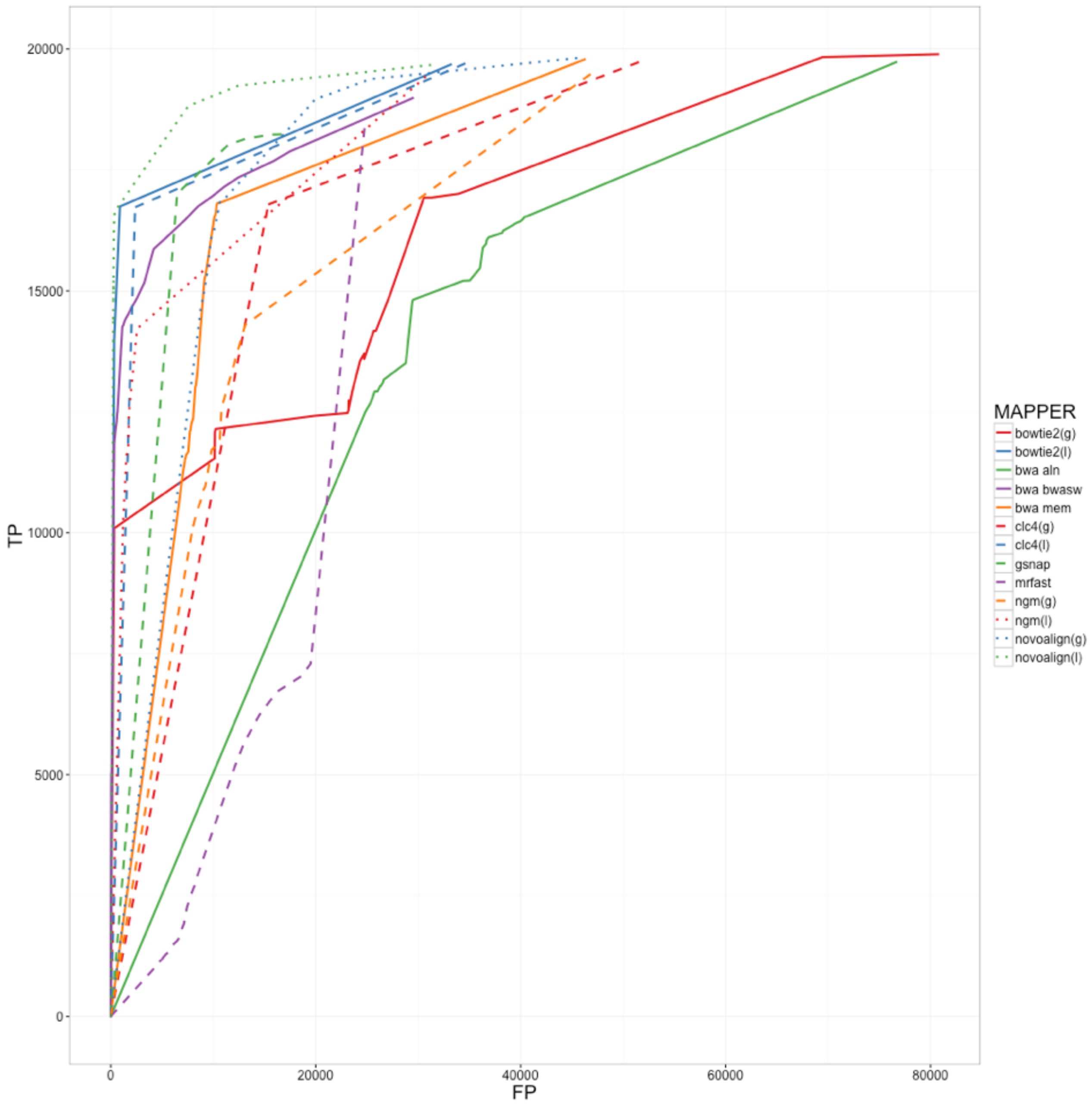

Supplement: Supplemental Material [file supp_g3.116.034488_FigureS11.pdf]
