# Supplementary material for: Suitability of Different Mapping Algorithms for Genome-Wide Polymorphism Scans with Pool-Seq Data
Source: G3 (Bethesda). 2016 Sep 9;6(11):3507–15. doi: 10.1534/g3.116.034488 (PMC5100849; doi:10.1534/g3.116.034488)
Supplement: Supplemental Material [file supp_g3.116.034488_TableS1.pdf]

Table 1: Effect of quality filtering. Filtering reads with a low mapping quality (<20) and reads not mapped as proper pairs substantially reduces the number of false positives (FP), improves allele frequency estimates (f) and reduces the number of outlier loci having highly inaccurate allele frequencies (OL; $f < 0.1$ or $f > 0.9$). However quality filtering also reduces the number of true positives (TP). ufi.: unfiltered, fi.: filtered

	TP		FP		f		OL	
	ufi.	fi.	ufi.	fi.	ufi.	fi.	ufi.	fi.
bowtie(g)	19892	12649	33034	0	0.585	0.556	805	367
bwa aln	19847	15822	31593	5	0.583	0.501	730	1
clc4(g)	19885	16667	32747	135	0.586	0.504	805	2
mrfast	18011	1277	8373	256	0.550	0.083	108	1109
ngm(g)	19111	10536	21711	28	0.572	0.495	547	10
novoalign(g)	19859	16508	30289	20	0.583	0.501	738	1
segemehl	19984	19984	62078	62078	0.605	0.605	1214	1214
bowtie(l)	19878	11078	31743	0	0.599	0.597	850	599
bwa bwasw	18813	14099	12462	0	0.585	0.524	209	2
bwa mem	15229	11418	8	0	0.501	0.502	4	7
clc4(l)	19878	16642	31878	62	0.594	0.513	830	6
gsnap	18003	16621	8112	250	0.560	0.520	117	5
ngm(l)	19092	10458	21378	0	0.580	0.504	555	13
novoalign(l)	19821	16446	28610	19	0.601	0.523	763	3
