# Supplementary material for: Suitability of Different Mapping Algorithms for Genome-Wide Polymorphism Scans with Pool-Seq Data
Source: G3 (Bethesda). 2016 Sep 9;6(11):3507–15. doi: 10.1534/g3.116.034488 (PMC5100849; doi:10.1534/g3.116.034488)
Supplement: Supplemental Material [file supp_g3.116.034488_TableS2.pdf]

Table 2: Suitability of mapping algorithms for performing genome wide polymorphism scans with Pool-Seq data. Ideally, a mapping algorithm should enable to identify all true positive SNPs (TP; 19.999 were simulated) and to estimate the allele frequencies accurately (μ_f average frequency of the reference allele; all SNPs were simulated with $f = 0.5$) while avoiding the identification of false positive SNPs (FP) and extreme outlier SNPs, with highly inaccurate allele frequency estimates (OL; $f > 0.9$ or $f < 0.1$). We tested the algorithm with three different data sets. best case: 2x100bp paired ends with an insert size of 100±0bp, indel - insert size: 2x100bp paired ends with an insert size of 100±40bp and indels between the SNPs, indel - error rate: 2x100bp paired ends with an insert size of 100±0bp, indels between the SNPs and an error rate of 5%.

	best case				indel - insert size				indel - error rate			
algorithm	TP	FP	μ_f	OL	TP	FP	μ_f	OL	TP	FP	μ_f	OL
bowtie2(g)	12649	0	0.556	367	12561	23222	0.576	642	10461	423k	0.855	2940
bwa aln	15822	5	0.501	1	15978	36634	0.538	184	14980	487k	0.583	189
clc4(g)	16667	135	0.504	2	16787	15376	0.511	155	16655	1527k	0.504	142
mrfast	1277	256	0.083	1109	1852	7038	0.120	1510	14920	661k	0.778	1032
ngm(g)	10536	28	0.495	10	10337	8290	0.529	134	9735	802k	0.521	87
novoalign(g)	16508	20	0.501	1	16630	10457	0.508	124	16482	1506k	0.507	148
segemehl	19984	62k	0.605	1214	19880	96448	0.610	1367	19987	1957k	0.640	1304
bowtie2(l)	11078	0	0.597	599	11290	281	0.616	846	11193	855k	0.534	97
bwa bwasw	14099	0	0.524	2	14259	1134	0.583	73	13807	1101k	0.645	219
bwa mem	11418	0	0.502	7	16558	10177	0.509	117	14451	1313k	0.349	3179
clc4(l)	16642	62	0.513	6	16730	2388	0.519	96	16606	1514k	0.516	155
gsnap	16621	250	0.520	5	17034	6607	0.526	65	17267	1491k	0.548	221
ngm(l)	10458	0	0.504	13	10186	1208	0.537	95	9610	775k	0.526	85
novoalign(l)	16446	19	0.523	3	16504	361	0.526	18	16396	1483k	0.527	168
