# Supplementary material for: Suitability of Different Mapping Algorithms for Genome-Wide Polymorphism Scans with Pool-Seq Data
Source: G3 (Bethesda). 2016 Sep 9;6(11):3507–15. doi: 10.1534/g3.116.034488 (PMC5100849; doi:10.1534/g3.116.034488)
Supplement: Supplemental Material [file supp_g3.116.034488_TableS3.pdf]

Table 3: Suitability of different mapping algorithms for performing genome wide polymorphism scans with unfiltered Pool-Seq data. Ideally, a mapping algorithm should enable to identify all true positive SNPs (TP; 19.999 were simulated) and to estimate the allele frequencies accurately (μ_f average frequency of reference allele; all SNPs were simulated with $f = 0.5$) while avoiding the identification of false positive SNPs (FP) and extreme outlier SNPs, with highly inaccurate allele frequency estimates (OL; $f > 0.9$ or $f < 0.1$). We tested the algorithm with three data sets; i) 2x100bp paired ends with an insert size of 100±0bp (best case) ii) 2x100bp paired ends with an insert size of 100±40bp and indels between the SNPs (indel - insert size) iii) 2x100bp paired ends with an insert size of 100±0bp, indels between the SNPs and an error rate of 5% (indel - error rate).

	best case				indel - md40				indel - error 5%			
	TP	FP	μ_f	OL	TP	FP	μ_f	OL	TP	FP	μ_f	OL
bowtie2(g)	19892	33034	0.585	805	19894	80826	0.592	1102	19960	1871960	0.654	1039
bwa aln	19847	31593	0.583	730	19740	76742	0.612	1248	17622	697699	0.615	1527
clc4(g)	19885	32747	0.586	805	19789	52261	0.592	981	19995	1968772	0.579	859
mrfast	18011	8373	0.550	108	18378	24773	0.565	265	17703	972556	0.807	2286
ngm(g)	19111	21711	0.572	547	19596	47559	0.591	937	19860	1932337	0.574	834
novoalign(g)	19859	30289	0.583	738	19821	45808	0.590	952	19981	1965562	0.582	827
segemehl	19984	62078	0.605	1214	19880	96448	0.610	1367	19987	1957388	0.640	1304
bowtie2(l)	19878	31743	0.599	850	19689	33282	0.605	944	19968	1933247	0.618	1062
bwa bwasw	18813	12462	0.585	209	18996	29578	0.594	473	19163	1784552	0.592	488
bwa mem	15229	8	0.501	4	19796	46347	0.590	943	19954	1851564	0.488	601
clc4(l)	19878	31878	0.594	830	19720	34781	0.599	945	19991	1963638	0.589	917
gsnap	18003	8112	0.560	117	18242	17459	0.567	212	18491	1701568	0.583	428
ngm(l)	19092	21378	0.580	555	19507	31166	0.597	877	19849	1924997	0.584	913
novoalign(l)	19821	28610	0.601	763	19670	31265	0.605	857	19974	1954622	0.600	931
