# Supplementary material for: Suitability of Different Mapping Algorithms for Genome-Wide Polymorphism Scans with Pool-Seq Data
Source: G3 (Bethesda). 2016 Sep 9;6(11):3507–15. doi: 10.1534/g3.116.034488 (PMC5100849; doi:10.1534/g3.116.034488)
Supplement: Supplemental Material [file supp_g3.116.034488_TableS4.pdf]

Table 4: Comparison of allele frequency differences between simulated Pool-Seq data sets with different mapping algorithms. We simulated different paired end Pool-Seq libraries, mapped the reads and compared the allele frequencies between the libraries using F_{ST}. With this procedure we evaluated the sensitivity of the alignment algorithm to differences in the distance between paired ends (id), differences in the read length (rl) and differences in the error rates (e). As all libraries were derived from identical template sequences (templates with SNPs and indels) no significant allele frequency differences were expected ($F_{ST} = 0$). We estimated the number of true positive SNPs for which allele frequencies could be compared (TP) and the lowest F_{ST}-values in the 0.1% and 10% quantiles with the most differentiated SNPs. id100, rl100, e1%: 2x100bp paired ends, insert size 100 ± 20bp, error rate 1%; id300: 2x100bp paired ends, insert size 300 ± 60bp, error rate 1%; rl50: 2x50bp paired ends, insert size 100 ± 20bp, error rate 1%; e5%: 2x100bp paired ends, insert size 100 ± 20bp, error rate 5%

	id100 vs. id300			rl100 vs. rl50			e1% vs. e5%		
algorithm	TP	10%	0.1%	TP	10%	0.1%	TP	10%	0.1%
bowtie2(g)	12468	0.021	0.358	12474	0.027	0.351	11778	0.251	0.476
bwa aln	14128	0.008	0.334	14106	0.014	0.279	14860	0.078	0.486
clc4(g)	12415	0.006	0.286	11067	0.008	0.201	12664	0.004	0.115
mrfast	10752	0.019	0.46	9712	0.055	0.500	11704	0.212	0.603
ngm(g)	8463	0.006	0.143	7122	0.030	0.468	7878	0.017	0.175
novoalign(g)	16093	0.004	0.289	15263	0.007	0.232	16415	0.003	0.081
segemehl	14097	0.005	0.223	13394	0.010	0.216	11550	0.011	0.136
bowtie2(l)	11158	0.031	0.359	11280	0.039	0.301	10663	0.024	0.668
bwa bwasw	13694	0.016	0.363	11433	0.262	0.456	13661	0.020	0.215
bwa mem	15990	0.005	0.290	15104	0.007	0.156	16288	0.004	0.075
clc4(l)	12415	0.006	0.286	11067	0.008	0.201	12664	0.004	0.115
gsnap	16363	0.007	0.231	12350	0.041	0.385	13615	0.044	0.372
ngm(l)	9665	0.006	0.142	9110	0.026	0.453	9079	0.018	0.167
novoalign(l)	16057	0.005	0.279	15191	0.009	0.243	16381	0.003	0.081
