# Supplementary material for: Suitability of Different Mapping Algorithms for Genome-Wide Polymorphism Scans with Pool-Seq Data
Source: G3 (Bethesda). 2016 Sep 9;6(11):3507–15. doi: 10.1534/g3.116.034488 (PMC5100849; doi:10.1534/g3.116.034488)
Supplement: Supplemental Material [file supp_g3.116.034488_TableS5.pdf]

Table 5: Comparison of allele frequency differences for false positive SNPs (FP) between simulated Pool-Seq data sets with different mapping algorithms. We simulated different paired end Pool-Seq libraries, mapped the reads and compared the allele frequencies between the libraries using F_{ST}. With this procedure we evaluated the sensitivity of the alignment algorithm to differences in the distance between paired ends (id), differences in the read length (rl) and differences in the error rates (e). As all libraries were derived from an identical population (with SNPs and indels) no significant allele frequency differences were expected. We estimated the number of false positive SNPs for which allele frequencies could be compared (FP) and and the quantiles of most extreme F_{ST}-values for 0.1% and 10% of the SNPs. id100, rl100, e1%: 2x100bp paired ends, insert size 100 ± 20bp, error rate 1%; id300: 2x100bp paired ends, insert size 300 ± 60bp, error rate 1%; rl50: 2x50bp paired ends, insert size 100 ± 20bp, error rate 1%; e5%: 2x100bp paired ends, insert size 100 ± 20bp, error rate 5%

	id: 100 vs. 300			rl: 100 vs. 50			e: 1% vs. 5%		
	FP	10%	0.1%	FP	10%	0.1%	FP	10%	0.1%
bowtie2(g)	706k	0.008	0.085	640k	0.008	0.077	760k	0.028	0.098
bwa aln	968k	0.007	0.073	896k	0.008	0.091	1186k	0.022	0.091
clc4(g)	1026k	0.007	0.073	957k	0.008	0.083	1505k	0.021	0.068
mrfast	816k	0.008	0.081	736k	0.011	0.100	995k	0.023	0.095
ngm(g)	567k	0.007	0.053	552k	0.008	0.091	817k	0.023	0.101
novoalign(g)	1062k	0.007	0.068	992k	0.008	0.083	1507k	0.020	0.065
segemehl	1285k	0.006	0.067	1238k	0.006	0.069	1416k	0.021	0.077
bowtie2(l)	573k	0.008	0.085	525k	0.008	0.063	885k	0.021	0.100
bwa bwasw	667k	0.008	0.082	368k	0.024	0.102	1143k	0.021	0.091
bwa mem	1048k	0.007	0.067	966k	0.008	0.083	1489k	0.020	0.063
clc4(l)	1026k	0.007	0.073	956k	0.008	0.083	1505k	0.021	0.068
gsnap	995k	0.007	0.081	661k	0.012	0.111	1118k	0.025	0.150
ngm(l)	535k	0.007	0.053	514k	0.008	0.088	799k	0.022	0.100
novoalign(l)	985k	0.007	0.067	883k	0.008	0.083	1493k	0.019	0.058
