# Supplementary material for: Suitability of Different Mapping Algorithms for Genome-Wide Polymorphism Scans with Pool-Seq Data
Source: G3 (Bethesda). 2016 Sep 9;6(11):3507–15. doi: 10.1534/g3.116.034488 (PMC5100849; doi:10.1534/g3.116.034488)
Supplement: Supplemental Material [file supp_g3.116.034488_TableS6.pdf]

Table 6: Comparison of allele frequency differences between real Pool-Seq data sets with different mapping algorithms. We compared allele frequencies between two paired end libraries with different read length and insert size that were prepared from the same genomic DNA (pooled *D. simulans* flies). We determined the number of SNPs for which allele frequencies could be compared (c) and the lowest F_{ST}-values in different quantiles with the most differentiated SNPs. mrfast generated an invalid output file with these data (an uniform read length was reported despite these reads having varying read lengths).

algorithm	c	10%	1%	0.10%	0.01%	0.001%
bowtie2(g)	4914k	0.074	0.154	0.274	0.429	0.651
bwa aln	5030k	0.064	0.137	0.253	0.437	0.660
clc4(g)	6610k	0.064	0.139	0.265	0.464	0.692
mrfast	na	na	na	na	na	na
ngm(g)	8427k	0.068	0.142	0.263	0.447	0.685
novoalign(g)	4964k	0.057	0.125	0.228	0.373	0.578
segemehl	4866k	0.072	0.151	0.285	0.492	0.750
bowtie2(l)	4745k	0.062	0.133	0.247	0.419	0.608
bwa bwasw	4531k	0.068	0.148	0.266	0.415	0.590
bwa mem	4786k	0.061	0.132	0.248	0.409	0.607
clc4(l)	4714k	0.064	0.143	0.275	0.464	0.670
gsnap	4897k	0.068	0.155	0.303	0.501	0.693
ngm(l)	5050k	0.064	0.143	0.283	0.492	0.708
novoalign(l)	4387k	0.060	0.132	0.238	0.387	0.570
