# Supplementary material for: Suitability of Different Mapping Algorithms for Genome-Wide Polymorphism Scans with Pool-Seq Data
Source: G3 (Bethesda). 2016 Sep 9;6(11):3507–15. doi: 10.1534/g3.116.034488 (PMC5100849; doi:10.1534/g3.116.034488)
Supplement: Supplemental Material [file supp_g3.116.034488_TableS7.pdf]

Table 7: Comparision of allele frequency differences using the same sequencing reads, but mapped as paired ends and single ends. The allele frequency differences between samples were assessed with F_{ST}. We estimated the number of true positive SNPs for which allele frequencies could be compared (TP; 19.999 were simulated) and the quantiles of most extreme F_{ST}-values for 0.1% and 10% of the SNPs. Paired end reads were simulated from a population with SNPs and indels: 2x100bp, insert size 100 ± 20bp, error rate 1%;

algorithm	FP	10%	0.1%
bowtie2(g)	11484	0.062	0.374
bwa aln	12361	0.009	0.350
clc4(g)	10396	0.002	0.245
mrfast	8930	0.025	0.379
ngm(g)	8267	0.000	0.016
novoalign(g)	13965	0.002	0.261
segemehl	12775	0.033	0.185
bowtie2(l)	10639	0.081	0.369
bwa bwasw	12660	0.022	0.537
bwa mem	11630	0.010	0.391
clc4(l)	10396	0.002	0.245
gsnap	13374	0.004	0.268
ngm(l)	9687	0.000	0.013
novoalign(l)	13954	0.002	0.256
