# Supplementary material for: Suitability of Different Mapping Algorithms for Genome-Wide Polymorphism Scans with Pool-Seq Data
Source: G3 (Bethesda). 2016 Sep 9;6(11):3507–15. doi: 10.1534/g3.116.034488 (PMC5100849; doi:10.1534/g3.116.034488)
Supplement: Supplemental Material [file supp_g3.116.034488_TableS8.pdf]

Table 8: Reduction of mapping artifacts by the intersection of mapping algorithms using the 0.001% quantile. Two Illumina paired end data sets with different insert sizes and read length were derived from pooled genomic DNA (natural *D. simulans* population) and mapped to the reference genome. Allele frequency differences between the libraries were computed using Fisher's exact test [-log(p-value) = *fet-value*]. To test which combination of alignment algorithms most efficiently reduces outlier peaks we intersected all pairwise combinations of alignment algorithms, i.e. we use SNPs identified with both algorithms and use the lowest *fet-value* found with any of the two algorithms. Below the diagonal we report the number of SNPs (in million) common to both algorithms. For comparision, the number of SNPs identified with a single mapping algorithm are shown next to the list of mappers on the left side. Above the diagonal we report the lowest *fet-value* among the 0.001% most differentiated SNPs (around 40 SNPs). For comparision, the corresponding *fet-values* obtained with a single mapping algorithm are shown next to the list of mappers on the upper side. We marked the five best combinations yielding the least pronounced outlier loci (green).

	bowtie2(g) 10.8	bwa aln 17.73	clc4(g) 15.81	ngm(g) 16.05	novoalign(g) 20.92	segemehl 21.44	bowtie2(l) 16.33	bwa bwasw 8.57	bwa mem 17.52	clc4(l) 11.6	gsnap 25.25	ngm(l) 11.87	novoalign(l) 17.24
bowtie2(g) 4.91		6.63	5.79	5.38	6.05	6.65	6.20	5.34	6.51	5.34	5.64	5.02	5.51
bwa aln 5.03	4.27		6.09	5.49	6.78	5.95	6.06	5.92	7.31	5.94	5.73	5.63	6.47
clc4(g) 6.61	4.10	4.27		9.07	7.81	7.12	5.52	5.38	7.89	9.68	7.39	5.72	7.76
ngm(g) 8.43	4.18	4.29	5.94		5.63	6.27	5.15	5.18	5.48	6.11	5.96	9.14	5.33
novoalign(g) 4.96	4.19	4.51	4.21	4.27		9.35	6.54	5.45	12.68	8.50	8.62	5.42	13.68
segemehl 4.87	4.05	3.91	3.97	4.12	3.92		8.26	5.49	9.36	7.71	7.62	5.46	7.72
bowtie2(l) 4.75	4.09	4.45	4.17	4.21	4.49	3.85		5.37	6.54	5.61	8.41	5.28	6.79
bwa bwasw 4.53	4.07	4.23	3.96	4.01	4.32	3.81	4.33		6.29	6.05	5.38	5.15	5.56
bwa mem 4.79	4.18	4.45	4.14	4.19	4.68	3.90	4.46	4.36		9.33	6.52	5.47	12.06
clc4(l) 4.71	4.01	4.32	4.35	4.20	1.48	3.80	4.37	4.21	4.28		5.63	6.67	7.91
gsnap 4.9	4.12	4.38	4.15	4.20	4.47	3.90	4.47	4.36	4.44	4.34		5.76	6.94
ngm(l) 5.05	4.15	4.45	4.40	4.60	4.45	3.96	4.51	4.32	4.40	4.54	4.48		5.17
novoalign(l) 4.39	3.84	4.12	3.88	3.85	4.35	3.62	4.20	4.13	4.28	4.07	4.19	4.13	
