# Supplementary material for: Suitability of Different Mapping Algorithms for Genome-Wide Polymorphism Scans with Pool-Seq Data
Source: G3 (Bethesda). 2016 Sep 9;6(11):3507–15. doi: 10.1534/g3.116.034488 (PMC5100849; doi:10.1534/g3.116.034488)

Figure 2: Mapping quality distribution for different mappers. Uniformly distributed paired end reads (2x100bp) with an insert size of 100±0bp and an error rate of 0% were simulated from sequences with SNPs every 100bp and aligned. Mapping qualities were obtained from the resulting bam files.

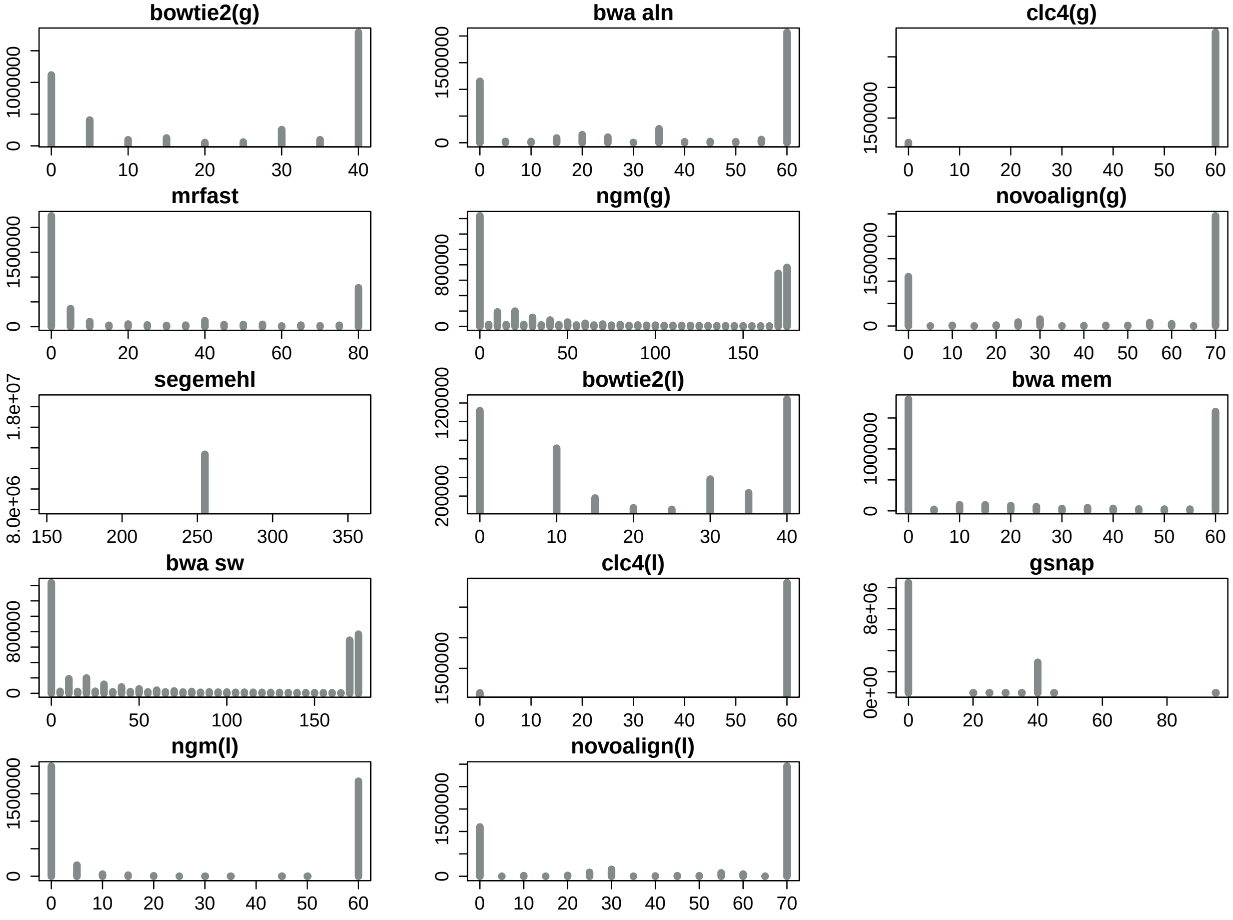

Supplement: Supplemental Material [file supp_g3.116.034488_FigureS2.pdf]
