# Supplementary material for: Suitability of Different Mapping Algorithms for Genome-Wide Polymorphism Scans with Pool-Seq Data
Source: G3 (Bethesda). 2016 Sep 9;6(11):3507–15. doi: 10.1534/g3.116.034488 (PMC5100849; doi:10.1534/g3.116.034488)

Figure 5: Manhattan plots showing the differentiation between a *D. melanogaster* population inoculated with C-virus for 20 generations and a control population (data from Martins et al., 2014). The three most differentiated SNPs identified by Martins et al. (2014) are shown in red [3L:7350895, X:7984325, X:7984449; coordinates were converted from assembly R5 to R6 using liftOver]. Four replicates of a population inoculated with C-virus (VirSys) and four replicates of a control population (ContSys) were mapped with bwa aln (0.6.2) and novoalign(g), and the differentiation between treatments was assessed for each SNP with a Cochran-Mantel-Haenszel test. Results were intersected by using for each SNP the least significant differentiation between the two mappers (bottom panel). This demonstrates that intersecting the results of two alignment algorithm preserves the targets of selection.

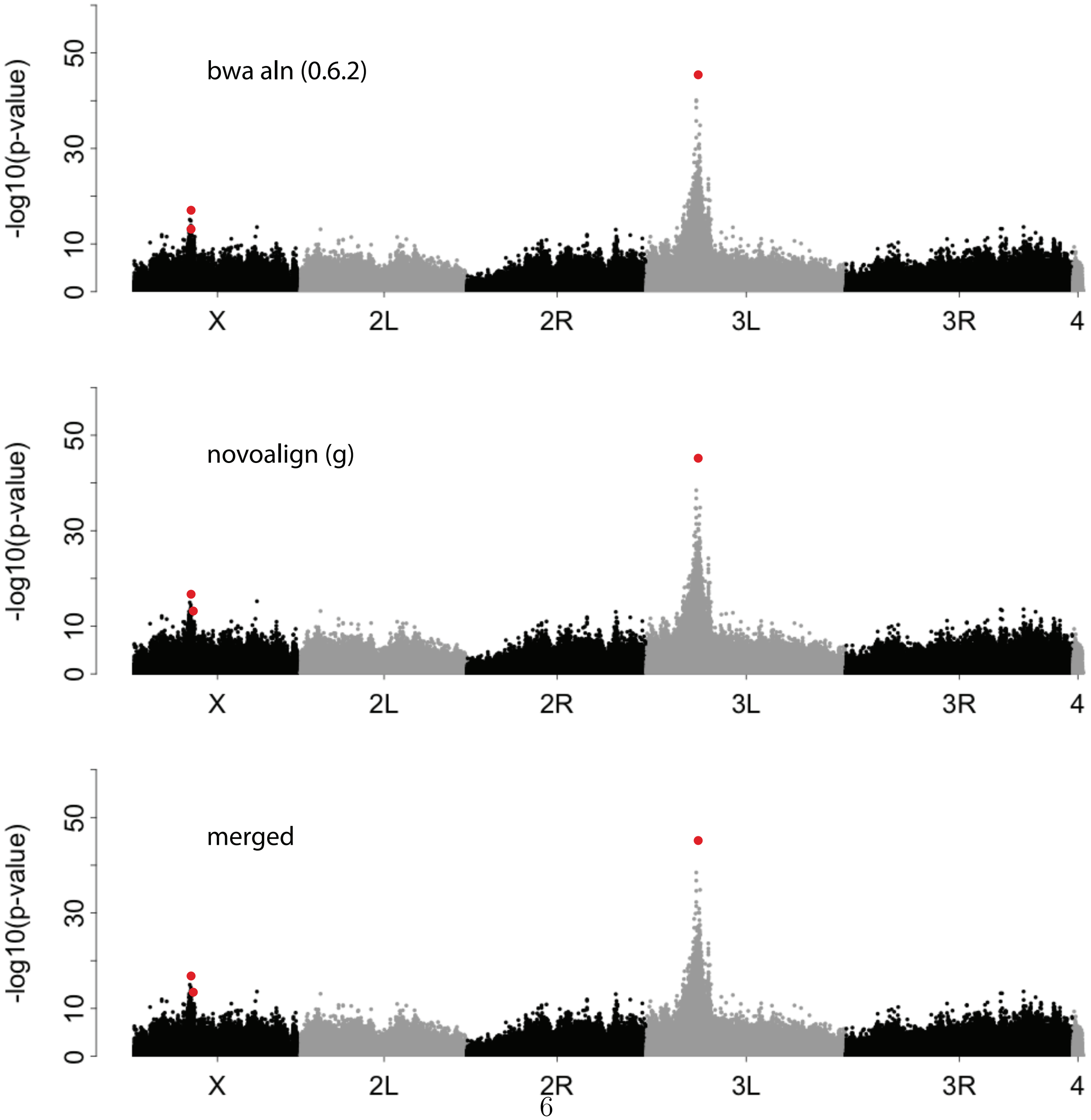

Supplement: Supplemental Material [file supp_g3.116.034488_FigureS5.pdf]
