# Supplementary material for: Suitability of Different Mapping Algorithms for Genome-Wide Polymorphism Scans with Pool-Seq Data
Source: G3 (Bethesda). 2016 Sep 9;6(11):3507–15. doi: 10.1534/g3.116.034488 (PMC5100849; doi:10.1534/g3.116.034488)
Supplement: Supplemental Material [file supp_g3.116.034488_TableS10.pdf]

Table 10: Comparing allele frequency estimates between samples mapped with different alignment algorithms. We simulated different paired end read data sets, mapped the reads with different mappers (sample indicated on the left with algorithm1 and right sample with algorithm2) and compared the allele frequencies using F_{ST}. We evaluated the effect of mixing alignment algorithm with samples having different inner distance between paired ends (id), different read lengths (rl) and different error rates (e). All libraries were derived from an identical population (with SNPs and indels) and therefore no significant allele frequency differences were expected. We estimated the number of true positive SNPs for which allele frequencies could be compared (TP) and the lowest F_{ST}-values in the 0.1% and 10% quantiles with the most differentiated SNPs. The effect of mixing algorithm was evaluated with respect to an unmixed mapping strategy, where we marked benchmarks that were either better (green) or worse (red) than both parent algorithm. The overall suitability of each combination was determined (count top - count worst; top ≥ 1; worst ≤ -1) and combinations of algorithms were marked accordingly. This illustrates that mixing algorithm, different parameters of algorithm or even different versions of algorithm mostly leads to worse results than an consistent alignment strategies that relies on the same approach for all samples. bwa aln* optimized parameters (-o 1 -l 200 -n 0.01 -d 12 -e 12) according to Kofler et al. (2011). id100, rl100, e1%: 2x100bp paired ends, insert size 100 ± 20bp, error rate 1%; id300: 2x100bp paired ends, insert size 300 ± 60bp, error rate 1%; rl50: 2x50bp paired ends, insert size 100 ± 20bp, error rate 1%; e5%: 2x100bp paired ends, insert size 100 ± 20bp, error rate 5%

		id100 vs. id300			rl100 vs. rl50			e1% vs. e5%		
algorithm1	algorithm2	TP	10%	0.1%	TP	10%	0.1%	TP	10%	0.1%
bowtie2(g)	bowtie2(g)	12468	0.021	0.358	12474	0.027	0.351	11778	0.251	0.476
novoalign(g)	novoalign(g)	16093	0.004	0.289	15263	0.007	0.232	16415	0.003	0.081
bowtie2(g)	novoalign(g)	12954	0.046	0.416	12832	0.044	0.411	12967	0.044	0.395
bowtie2(g)	bowtie2(g)	12468	0.021	0.358	12474	0.027	0.351	11778	0.251	0.476
bowtie2(l)	bowtie2(l)	11158	0.031	0.359	11280	0.039	0.301	10663	0.024	0.668
bowtie2(g)	bowtie2(l)	11788	0.037	0.401	12015	0.069	0.403	10714	0.024	0.444
novoalign(g)	novoalign(g)	16093	0.004	0.289	15263	0.007	0.232	16415	0.003	0.081
novoalign(l)	novoalign(l)	16057	0.005	0.279	15191	0.009	0.243	16381	0.003	0.081
novoalign(g)	novoalign(l)	16076	0.006	0.274	15200	0.011	0.261	16393	0.005	0.113
bwa aln (074)	bwa aln (074)	15385	0.008	0.333	14393	0.014	0.279	15342	0.081	0.472
bwa mem (074)	bwa mem (074)	15990	0.005	0.290	15104	0.007	0.156	16288	0.004	0.075
bwa aln (074)	bwa mem (074)	15596	0.011	0.373	14886	0.010	0.338	15811	0.009	0.392
bwa aln (074)	bwa aln (074)	15385	0.008	0.333	14393	0.014	0.279	15342	0.081	0.472
bwa aln* (074)	bwa aln* (074)	15276	0.005	0.280	14246	0.008	0.187	15567	0.036	0.203
bwa aln (074)	bwa aln* (074)	15332	0.009	0.338	14241	0.012	0.209	15679	0.027	0.387
bwa aln (058)	bwa aln (058)	14141	0.008	0.343	14116	0.014	0.279	14859	0.078	0.486
bwa aln (074)	bwa aln (074)	15385	0.008	0.333	14393	0.014	0.279	15342	0.081	0.472
bwa aln (058)	bwa aln (074)	15092	0.008	0.349	14109	0.014	0.292	15043	0.082	0.472
