# Supplementary material for: Suitability of Different Mapping Algorithms for Genome-Wide Polymorphism Scans with Pool-Seq Data
Source: G3 (Bethesda). 2016 Sep 9;6(11):3507–15. doi: 10.1534/g3.116.034488 (PMC5100849; doi:10.1534/g3.116.034488)

Figure 4: Example of an outlier peak identified with novoalign but not with bwa. Log transformed fisher exact test p-valules (fet) indicate the significance allele frequency differences. The coverage and some aligned reads are shown for the two *D. simulans* libraries (read length 76bp and 120bp) aligned either with bwa or novoalign. Data were visualized with IGV.

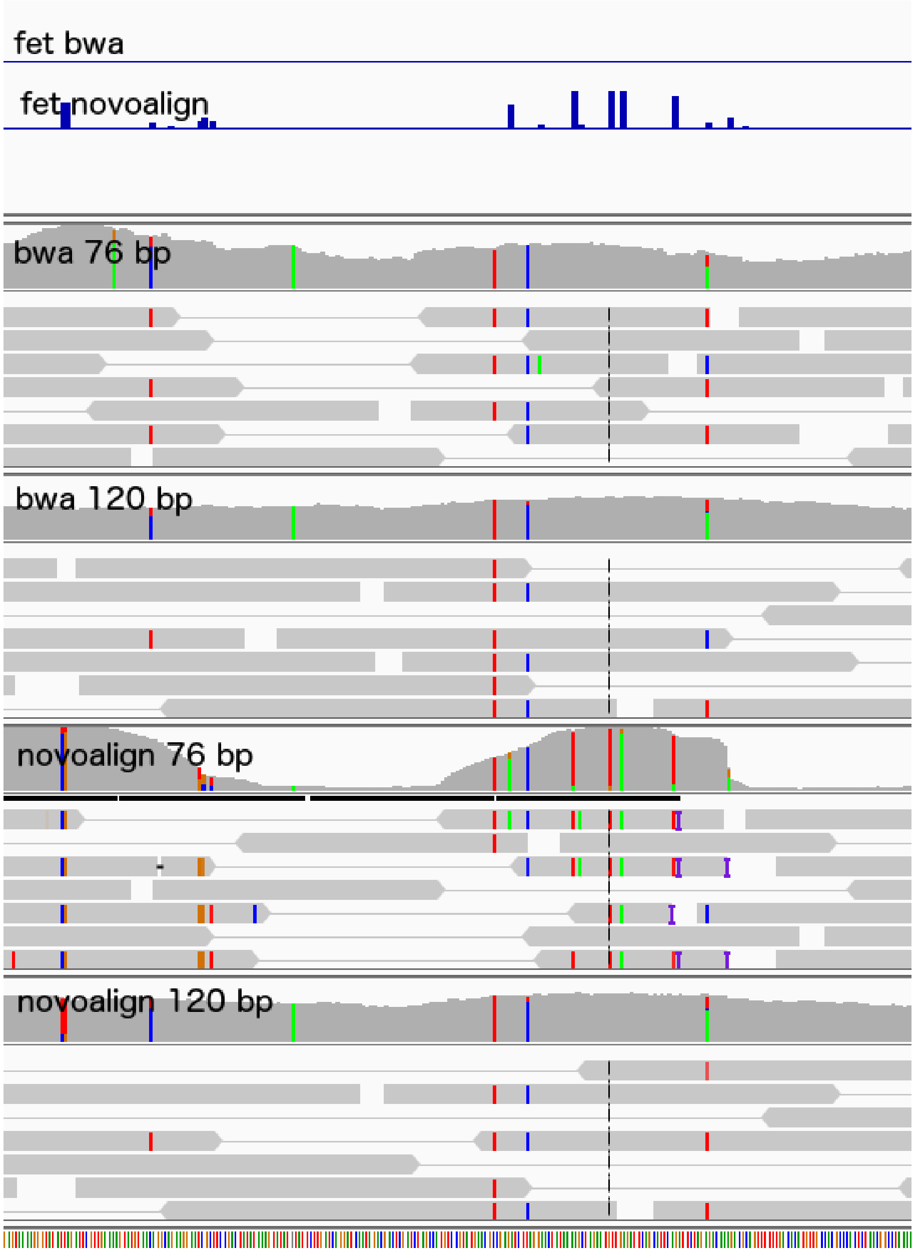

Supplement: Supplemental Material [file supp_g3.116.034488_FigureS4.pdf]
