# Supplementary material for: Suitability of Different Mapping Algorithms for Genome-Wide Polymorphism Scans with Pool-Seq Data
Source: G3 (Bethesda). 2016 Sep 9;6(11):3507–15. doi: 10.1534/g3.116.034488 (PMC5100849; doi:10.1534/g3.116.034488)

Figure 7: Manhattan plots for reads aligned with bowtie2(g) (top panel), novoalign(l) (middle panel) and for the intersection of the mapping results (bottom panel). Two Illumina paired-end sequencing libraries with different reads length and insert sizes were prepared from identical genomic DNA (a pool of a natural *D. simulans* populations).

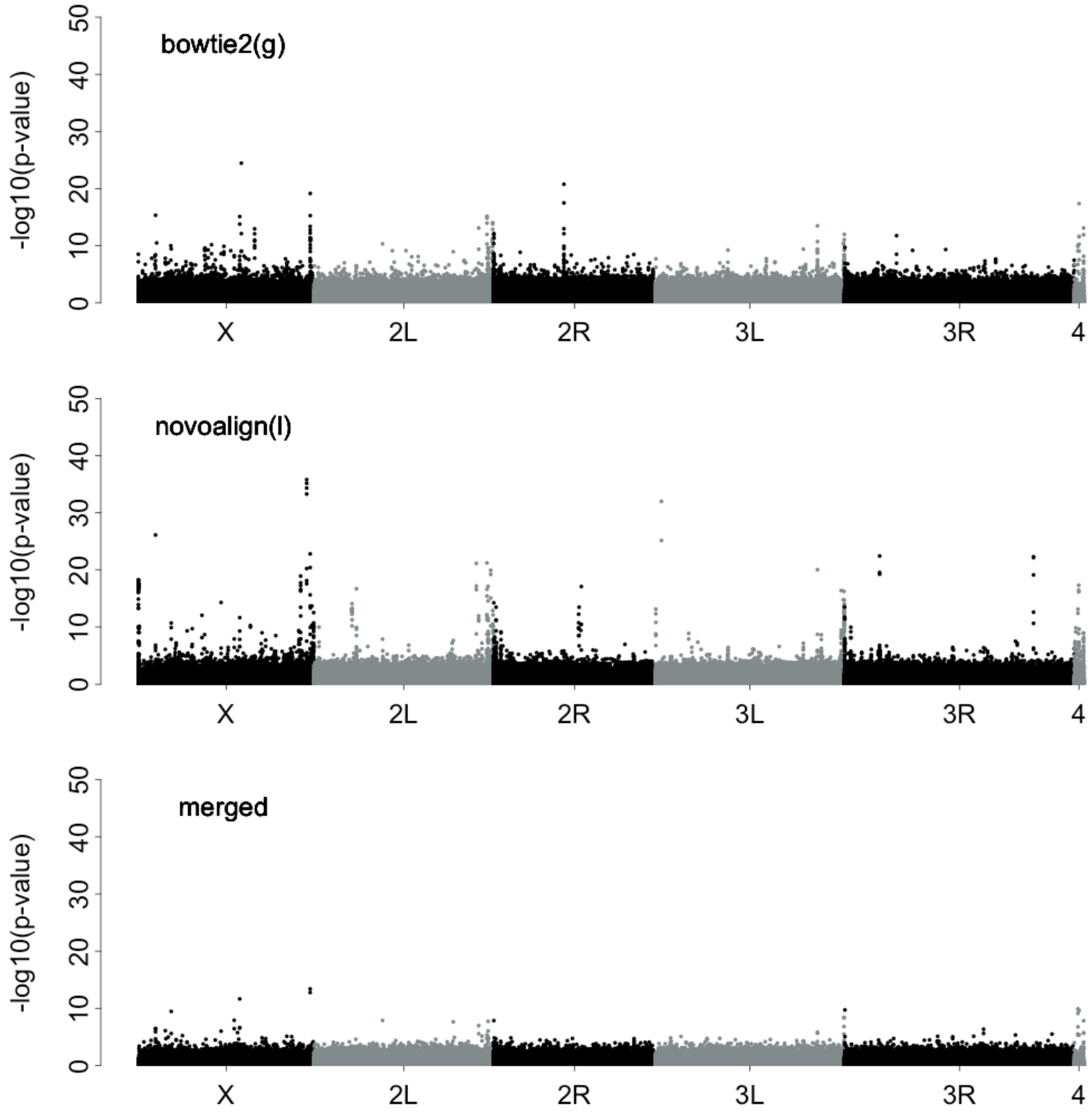

Supplement: Supplemental Material [file supp_g3.116.034488_FigureS7.pdf]
