# Supplementary material for: Suitability of Different Mapping Algorithms for Genome-Wide Polymorphism Scans with Pool-Seq Data
Source: G3 (Bethesda). 2016 Sep 9;6(11):3507–15. doi: 10.1534/g3.116.034488 (PMC5100849; doi:10.1534/g3.116.034488)

Figure 9: Manhattan plots for a comparision of two of paired end data sets having similar read length and insert size. Two lanes of a Illumina paired-end sequencing libraries (2x76bp) were sequenced from an identical library, reads were mapped to the reference genome and significant differences in allele frequencies between the two lanes were computed (Fisher's exact test). Reads were aligned, either with bwa aln (top panel), novoalign (middle panel) or the intersection of the two mapping results was used (bottom panel).

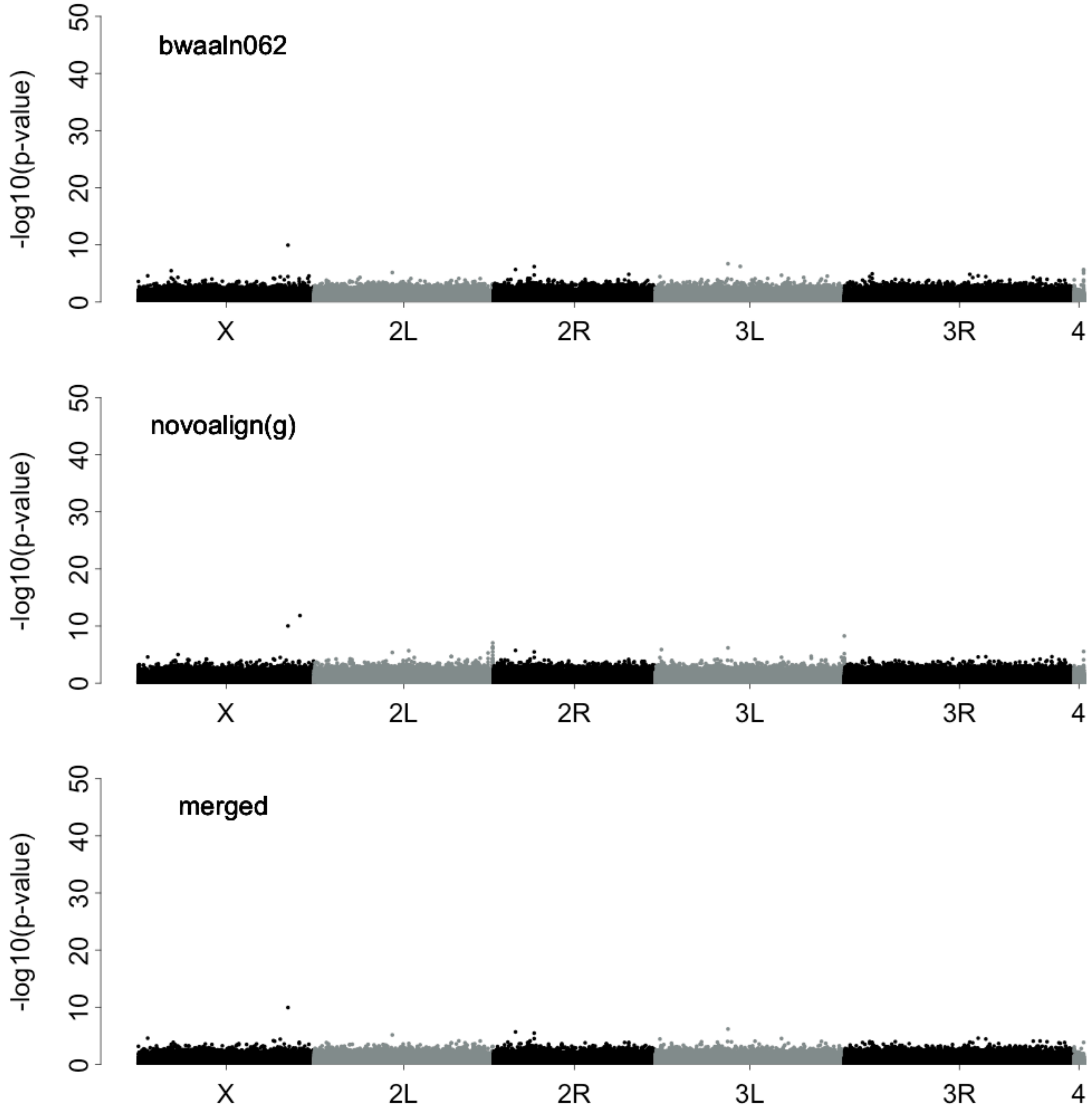

Supplement: Supplemental Material [file supp_g3.116.034488_FigureS9.pdf]
